# Socioeconomic inequalities in health: individual or area level; does it matter?

**DOI:** 10.1186/1471-2458-12-171

**Published:** 2012-03-08

**Authors:** B Galobardes

**Affiliations:** 1 School of Social and Community Medicine, University of Bristol, Bristol, UK

**Keywords:** Health inequalities, Individual level, Area-based, Socioeconomic position, Cardiovascular disease, Risk factors, Trends, Indicators

## Abstract

In the last decades we have accumulated substantial knowledge about the risk factors that lead to cardiovascular disease. Despite this progress, in this issue of BMC Public Health we learn that little improvement has been made towards reducing inequalities in these risk factors in the UK. Characterizing changes over time can help understanding the mechanisms that underpin health inequalities. These pathways are complex and operate at different levels, from the individual to the context where someone lives. In this commentary I highlight some of the issues and uncertainties that may arise when individual and area level measures are used indistinctively.

## 

In this issue, Scholes et al. present an interesting and thorough investigation of trends in inequalities in cardiovascular disease (CVD) risk factors in the UK from 1994 to 2008 [1]. This research shows decreasing trends in prevalence of smoking, raised blood pressure and raised cholesterol but increasing prevalence of obesity and diabetes in the last 15 years. We also learn that in terms of inequalities, although the picture is complex, little progress has been made towards reducing them.

There is one aspect in this work that puzzled me. Measurement issues have always been central to epidemiology and they equally apply to health inequalities [2,3]. If inequalities in a health outcome exist, most indicators of socioeconomic position (SEP) will capture them. If no measure of SEP is available in a dataset, we can be very creative and find a proxy that might capture this construct. That is because SEP is so embedded in our societies that it manifests in most, if not all, aspects of our lives. Researchers have indeed been very inventive and there are examples of proxies such as the luminosity of a country [4], the height of commemorative obelisks in burials [5] or the number of children a person has [6] to name a few. Very often researchers have turned to area-based measures by assigning to individuals the "average" SEP of a relatively small area [7]. What I found intriguing in Scholes et al.'s work is that the opposite is true. For those not familiar with the Health Survey of England (HSE), occupational class and educational attainment are measured every year. Housing tenure and income are available for most years. Why would the authors go through the trouble to link postcodes and assign area proxies to participants for whom they had individual-level measures? And, more importantly, does it matter?

The answer to the first question is unclear to me. The authors discuss the choice of SEP indicator in the paper. First, they point to research of area level characteristics, which shows that these have an independent role over and above that of individual-level measures. The statement is correct and indeed "place effects" is a very interesting field of research. However, area-level characteristics are relevant when that is the level of enquiry and importantly, require area-level methods to correctly identify and interpret them. This is not the case in the work presented here as the aim was clearly at the individual level. Secondly, the authors argue that area-based measures are particularly useful "proxy measures of individual social position in older age groups" (my underlining) [1]. Again, a potentially valid point, which would have been interesting to test in this sample would the sample have been of an old age. This is not the case for the HSE. Thirdly, the authors argue that using an area-based indicator enables them to examine whether the trends and inequalities in risk factors could help explain trends and inequalities in coronary heart disease (CHD) mortality. Given that no analysis of mortality trends in CHD are presented or discussed in this paper I fail to understand why this could be a reason to use area proxies rather than the readily available and more appropriate individual-level indicators. I don't think we can speculate how readers will use the information presented in this paper. In my opinion, a published paper should stand alone and should use the most appropriate data and methods available, independently of future analysis or assumptions of how it will be used.

But, does it really matter? Yes, I think it does. Conceptually, area and individual socioeconomic characteristics are not the same constructs. A wealth of research shows, including the articles cited by the authors, that they should not be used indistinctively [7-10]. It also matters in practice. Using area-based SEP may underestimate the association between individual SEP and the health outcome due to misclassification of the individual SEP level [8]. On the other hand, if area characteristics independently influence health outcomes, the association between area measures and health will overestimate the individual level [8]; that is also the case if the area-based measure captures a broader construct than the individual one [7]. We do not know if, in which direction or how much the estimates in this work are actually biased. But, this uncertainty was avoidable. Another problem, acknowledged by the authors, is that the 2007 Index of Multiple Deprivation was used to assign individual SEP to all survey years. Whether this measure was capturing area, let alone individuals, SEP equally throughout the study period is questionable. The magnitude of this bias may be differential by year, a potential problem when analysing trends. Conceptually it is also a problem that, for some years, the outcomes occurred before the exposure took place. This potential bias was unnecessary; it was created by the choice of SEP indicator. While in other research settings these might be unavoidable limitations, this was not the case in this study.

In summary, we should aim to use the appropriate measures for the level of inquiry we are interested in. Using the best data available will further our knowledge and understanding of the magnitude and pathways through which inequalities result in ill health. Ultimately, it is this knowledge, and political willingness, what can help prevent them.

## Abbreviations

CVD: Cardiovascular disease; SEP: Socioeconomic position; HSE: Health survey of England; CHD: Coronary heart disease

## Competing interests

The author declares that she has no competing interests.

## Pre-publication history

The pre-publication history for this paper can be accessed here:

http://www.biomedcentral.com/1471-2458/12/171/prepub
